# Flexible use of post-saccadic visual feedback in oculomotor learning

**DOI:** 10.1167/jov.22.1.3

**Published:** 2022-01-07

**Authors:** Frauke Heins, Markus Lappe

**Affiliations:** 1 Institute for Psychology and Otto-Creutzfeldt Center for Cognitive and Behavioral Neuroscience, University of Muenster, Muenster, Germany

**Keywords:** saccade adaptation, saccadic eye movements, motor learning, post-saccadic feedback

## Abstract

Saccadic eye movements bring objects of interest onto our fovea. These gaze shifts are essential for visual perception of our environment and the interaction with the objects within it. They precede our actions and are thus modulated by current goals. It is assumed that saccadic adaptation, a recalibration process that restores saccade accuracy in case of error, is mainly based on an implicit comparison of expected and actual post-saccadic position of the target on the retina. However, there is increasing evidence that task demands modulate saccade adaptation and that errors in task performance may be sufficient to induce changes to saccade amplitude. We investigated if human participants are able to flexibly use different information sources within the post-saccadic visual feedback in task-dependent fashion. Using intra-saccadic manipulation of the visual input, participants were either presented with congruent post-saccadic information, indicating the saccade target unambiguously, or incongruent post-saccadic information, creating conflict between two possible target objects. Using different task instructions, we found that participants were able to modify their saccade behavior such that they achieved the goal of the task. They succeeded in decreasing saccade gain or maintaining it, depending on what was necessary for the task, irrespective of whether the post-saccadic feedback was congruent or incongruent. It appears that action intentions prime task-relevant feature dimensions and thereby facilitated the selection of the relevant information within the post-saccadic image. Thus, participants use post-saccadic feedback flexibly, depending on their intentions and pending actions.

## Introduction

Eye movements are not only essential for our visual perception, but also for our interaction with the environment in which we move. They precede many of our everyday actions and in this way also provide information about our intentions (Hayhoe & Ballard, 2005; Land & Hayhoe, 2001). Eye movements remain accurate under constantly changing conditions and across the lifespan (Munoz, Broughton, Goldring, & Armstrong, 1998; Warabi, Kase, & Kato, 1984) due to continuous recalibration. In particular, saccades, the rapid eye movements that align our fovea with the object of interest, require adjustment of the motor command because they are of such short duration that correction of their trajectory cannot be performed while they are in flight. Instead, post-saccadic feedback is required to make any necessary adjustments to restore or maintain accuracy (Collins & Wallman, 2012; Havermann & Lappe, 2010; Noto & Robinson, 2001; Wallman & Fuchs, 1998; Wong & Shelhamer, 2011). The adjustment of saccade amplitude, also termed saccade adaptation, is based on a comparison of the expected and actual sensory consequences of the eye movement, from which a motor error is postdicted (Masselink & Lappe, 2021). Saccadic adaptation then aims to minimize this postdicted motor error.

Saccade adaptation can be studied in the laboratory with an intra-saccadic step of the saccade target (McLaughlin, 1967), which typically goes unnoticed due to saccadic suppression of displacement (Bridgeman, Hendry, & Stark, 1975). After saccade landing, the visual image of the target is thus at an unexpected location on the retina and constitutes a post-saccadic error signal. If the intra-saccadic target displacement is repeated, the saccade amplitude is gradually adjusted to move the saccade landing position closer to the stepped position of the target and to reduce the error (McLaughlin, 1967).

Saccade adaptation was long assumed to be an automatic, low-level mechanism that restores saccade accuracy, but there is increasing evidence that suggests that task demands have not only a modulatory effect on saccade adaptation, but can even be a sufficient cause for an adjustment of eye movement behavior. For example, saccade adaptation can occur in the absence of a spatial prediction error and instead follow reinforcement (Madelain, Paeye, & Wallman, 2011). Schütz, Kerzel, and Souto (2014) showed that participants shorten and lengthen their saccade amplitude in the absence of any error if this increases their performance in a perceptual task, indicating that saccade motor performance might also be driven by current task goals. These adjustments of saccade amplitude that are presumably modulated by top-down processes resemble those following a bottom-up visual error. Both develop gradually over time, reach the same magnitude, and show persisting after effects (Madelain et al., 2011; Schütz et al., 2014; Schütz & Souto, 2015). Thus, they seem to represent not only a short-term strategic adjustment of the oculomotor behavior, but actual learning.

Further evidence for saccadic adaptation being moderated by top-down processes stems from studies that highlight a strong effect of target selection. Saccade adaptation is selective to the saccade target (Herman, Harwood, Wallman, & Madelain, 2010), it is unhampered by distractors (Madelain, Harwood, Herman, & Wallman, 2010; but see Khan, McFadden, Harwood, & Wallman, 2014) and it is specific to the position changes of the saccade goal (Madelain, Herman, & Harwood, 2013). Moreover, Wolf, Wagner, and Schütz (2019) demonstrated that target selection in the presence of multiple targets is driven by the behavioral goal as well as that the end point of the saccade can be adjusted to meet task demands. Thus, humans can voluntarily choose which target to adapt to. Humans can even voluntarily prevent their saccade amplitude from adapting to a visual error when this is necessary to achieve a task goal (Heins, Meermeier, & Lappe, 2019). These findings, taken together with further studies highlighting the importance of task demands (Schütz et al., 2014) and error evaluation (Wagner, Wolf, & Schütz, 2021) on saccadic adaptation or impending goal-directed motor actions on eye movements (Hayhoe, 2000; Land, Mennie, & Rusted, 1999), indicate that bottom-up and top-down signals converge to define the appropriate oculomotor behavior (for a review see Souto & Schütz, 2020).

Talking about saccade adaptation, one usually considers the impact of visual feedback, especially the post-saccadic position error that describes that the saccade goal does not appear at the expected position on the retina. Yet, a saccade target object does not have to be defined by its position only, because the visual scene contains more information than just the spatial position of objects. It provides, for example, surface feature information as shape and color; or information regarding the relative position of an object to other objects. In everyday life, we make eye movements to objects that we want to act upon and that are not defined by their position but by visible features, such as color or shape. This target object feature information, stored across saccades in visual working memory, contributes to target recognition and object correspondence, both of which are essential to link the pre- and post-saccadic images and create the impression of a stable and coherent visual world (Currie, McConkie, Carlson-Radvansky, & Irwin, 2000; Hollingworth, Richard, & Luck, 2008; Tas, Moore, & Hollingworth, 2012; for a review see Van der Stigchel & Hollingworth, 2018).

The recognition of the saccade target is also necessary to create a post-saccadic estimate of saccade accuracy and guide corrective saccades. Hollingworth et al. (2008) investigated the effect of incongruent sources of information in the post-saccadic image on object correspondence and corrective saccades (i.e. saccades that are made after a primary saccade to a specific target does not fully reach that target). They found that humans can use the different information sources flexibly to guide their oculomotor behavior. In their study, saccades were made toward a saccade target defined by its position or by its color. The scene changed during the primary saccade thus requiring a corrective secondary saccade. In different conditions with specific scene changes, the information sources in the post-saccadic visual feedback after the primary saccade were either congruent (i.e. indicating the same goal for the corrective eye movement), or they were incongruent (i.e. constituting conflicting information regarding the correct goal for the corrective saccade). In both cases, participants managed to perform their corrective saccades to the defined target object in the majority of trials. In this respect, the weighting of both sources of information was dependent on the task demands. Yet, if the two sources of information were incongruent (i.e. there was a conflict in the visual feedback), corrective saccades showed a longer latency and a higher error rate, suggesting an interference. The interference was stronger for incongruent position information than for incongruent color information, which is in line with the assumption that position information is the dominant information source behind saccade planning (Adler, Bala, & Krauzlis, 2002).

In the present study, we investigated if the different sources of information within the post-saccadic feedback cannot only be used flexibly for guiding correction of gaze, but also for adjusting the primary saccade amplitude. Thus, we conducted an experiment during which we defined the saccade goal either by its color or by its position within an object array. We manipulated the post-saccadic feedback and investigated amplitude adjustment as well as inhibition of amplitude adjustment in the presence of conflicting information sources and in a control condition with congruent information. We hypothesized that the task goal (i.e. making an eye movement toward either the object defined by its color or the object defined by its position), would influence the use of post-saccadic information sources for adaptation. We also hypothesized that adaptation can occur without a spatial error, if the task requires a change to saccade amplitude. Finally, we expected that incongruent information within the post-saccadic visual feedback would lead to interference (i.e. weaker adaptation or inhibition).

## Methods

### Sample

The sample consisted of 36 participants (23 women) aged between 19 and 48 years (*M* = 25.05, *SD* = 5.94). All participants had normal or corrected-to-normal vision and gave their informed consent in written form before participating in the study. They were compensated for their time with either course credit or 8 €/h.

### Experimental setup

The experiment was conducted in a dimly illuminated room in the Institute for Psychology of the University of Muenster. The participants sat 67 centimeters in front of an Eizo FlexScan 22-inch monitor (Eizo, Hakusan, Japan) running at a frame rate of 75 Hz with a screen resolution of 1152 × 870 pixels. Viewing was binocular while the right eye was recorded using the Eyelink 1000 eye tracker (SR Research, Ontario, Canada) at a sampling frequency of 1000 Hz. The experimental code was written in MATLAB 2018a (Mathworks, Natick, MA, USA) using the Psychophysics Toolbox extension (Brainard, 1997; Kleiner et al., 2007). A stable head position was ensured using a custom developed chin-forehead rest.

The experimental procedures were approved by the Ethics Committee of the Department of Psychology and Sport Science of the University of Muenster.

### Stimuli and procedure

**Figure 1.**
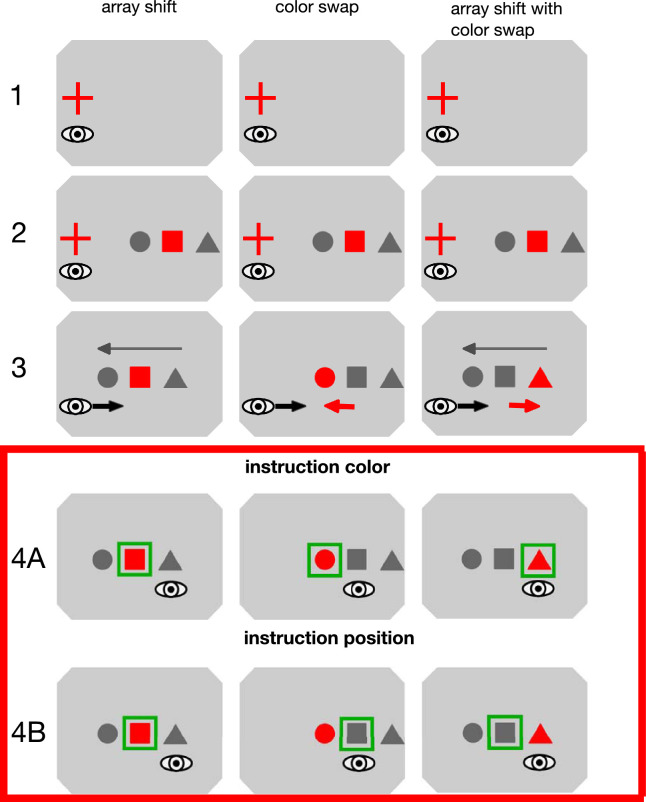
Trial layout for adaptation trials in the different feedback conditions, depicted for the instruction to look at the colored object (instruction color) and the instruction to look at the object at the position within the array that is initially indicated by the red color (instruction position). In all conditions, participants first looked at a fixation cross. Once a stable initial fixation had been detected, the object array appeared on the right side of the screen while the participants still fixated the fixation cross. After a time interval of 500 to 1000 ms had elapsed, the fixation cross was turned off and the subjects were to initiate a saccade to the defined saccade target. In the array shift condition, the object array shifted 2 degrees against saccade direction. The pre-saccadically colored object remained red. In the color swap condition, the object array maintained its position, but the initially colored object became grey and the object to the left turned red. In the array shift with color swap condition, the object array was shifted 2 degrees against the saccade direction. Simultaneously, the initially colored object became grey and the object to the right turned red. The green rectangle around the target object indicates the correct target object, depending on the instruction, and was not shown on the screen during the experiment. Stimuli are not drawn to scale.

Stimuli were presented on a grey background (8.01 cd/m^2^; Figure 1). At the beginning of each trial, a red fixation cross (0.6 degrees × 0.6 degrees) was displayed on the left side of the computer screen. Its position varied between trials on the horizontal (up to 2 degrees left or right) and the vertical axis (up to 1 degree up- or downward) in a counterbalanced manner to counteract the execution of a stereotyped saccade. Before the trial started, the subjects’ eye position had to be in a 2 × 2 deg fixation window around the fixation cross for at least 300 milliseconds. After detection of a valid, initial fixation, an array containing three objects was drawn on the screen. The objects were a square (1 × 1 degrees), an isosceles triangle (base: 1 degree; height: 1 degree) and a circle (1 degree diameter) spaced 2 degrees apart. The center of the object array was 10 degrees to the right of the fixation cross. Thus, the three objects appeared at distances of 8, 10, and 12 degrees to the fixation cross, respectively. The order of the objects within the object array varied between trials. One of the objects was colored red (2.75 cd/m^2^) upon appearance, the other objects were of darker grey color (3.56 cd/m^2^). Participants continued to look at the fixation cross for another 500 to 1000 ms until it was removed from the screen. If fixation was interrupted before the fixation cross was removed, a sinusoidal tone was played and the current trial was aborted and restarted. Subsequent events depended on the type of trial and the experimental condition.

### Conditions

To investigate whether subjects can flexibly use post-saccadic information sources to adjust their gaze behavior to meet task demands, we used two different instructions. With the instruction “color” (see Figure 1), we asked the participants to always look at the colored object within the object array and, if they did not foveate the colored object after their primary saccade, to correct their gaze toward the colored object, independent of its post-saccadic position within the object array. With the instruction “position” (see Figure 1), we asked the participants to always look at the object at the position within the array that was indicated by the red color before the primary saccade. For example, if the second object in the array was colored red before they performed an eye movement, then they were to direct their gaze toward the object at the second position in the array, irrespective of any change in color after the primary saccade. Participants were pseudorandomly assigned to one of those instructions.

Participants in in each instruction group performed three recording sessions with different post-saccadic feedback conditions. These were recorded with a minimum time interval of 72 hours between any two sessions in order to avoid carry-over from one recording session to the next. Each session lasted approximately 25 minutes.

In the array shift condition, the object array shifted 2 degrees to the left upon detection of the primary saccade. The object that was colored before the saccade remained red. Thus, the different sources of information within the post-saccadic visual input were congruent. Both the color instruction and the position instruction thus required participants to shorten their amplitude in order to meet the task demands (i.e. foveate the colored object or foveate the object at the predefined position within the array, respectively).

In the color swap condition, the object array remained in place but the originally colored object became grey and the object to the left was colored red once the saccade was initiated. Consequently, the saccade landed on the initially colored, now grey object. Following the color instruction, participants fulfilled the task demands only if they adjusted their saccade to the post-saccadic color information and ignored the position information. Following the position instruction, participants fulfilled the task demands only if they continued to perform a saccade that was not adjusted to the post-saccadic color information and instead continued to aim for the predefined position within the object array. Color and position information were incongruent in this condition.

In the array shift with color swap condition, the object array was shifted 2 degrees to the left upon saccade onset. Simultaneously a color swap took place (i.e. the originally colored object turned grey and the object to the right of it was colored red). In this way, the saccade landed on the red object despite the array shift. Following the color instruction, participants fulfilled the task demands only if they continued to perform a saccade that was not adjusted to the position error induced by the array shift. Following the position instruction, participants fulfilled the task demands only if they adjusted their saccade amplitude to the array shift and ignored the post-saccadic color information. Color and position information were incongruent in this condition. Figure 1 depicts the layout for the adaptation trials in all three conditions and indicates the typical early adaptation saccade landing position as well as the post-saccadic target object, depending on the instructions.

All conditions began with 20 no-feedback pre-adaptation trials during which the object array was turned off upon saccade onset. Those trials were included to assess the baseline state before the start of the adaptation procedure. The adaptation procedure consisted of 200 trials during which the respective post-saccadic feedback was presented to the participants. The adaptation phase was followed by further 20 no-feedback post-adaptation trials to assess the after-effect of learning. In all conditions, primary saccades were aimed toward all three objects within the object array during the pre-and post-adaptation trials. However, during the adaptation procedure, primary saccades were aimed at all three objects only in the array shift condition. This is due to the fact that it is not possible to color the object to the left of the first object within the object arrangement or the object to the right of the third object. Hence, during the adaptation phase of the condition array shift with color swap, only the first or second object was colored red before saccade onset; and during the condition color swap, only the second or third object was colored red before the saccade was initiated. It also follows that complete adaptation of the saccade amplitude to the intra-saccadic manipulation during the adaptation phase varies slightly among the three feedback conditions. If participants were to adapt completely the gain change would be –20.57% in the array shift condition, –22.50% in the array shift with color swap condition, and –18.33% in the color swap condition. However, humans usually adjust their saccade motor performance only incompletely during adaptation such that the slight difference between condition does not seem relevant (Miller, Anstis, & Templeton, 1981).

### Data analysis

Trials during which participants performed valid primary saccades were included in the data analysis. Valid primary saccades were defined by having an amplitude between 4 and 16 degrees, a duration of less than 100 ms, and a latency between 100 ms and three median absolute deviations from the median latency of the participant. Following these criteria, 85.20% of primary saccades were used for further analysis.

Because the required saccade amplitude was different for the three targets in the array und thus varied from trial to trial, the amplitude itself is not suitable to represent the learning process and to quantify the changes to saccade motor performance throughout the experiment. Instead, saccade gain G was used. It describes the ratio of the actual saccade amplitude and the distance the eye needed to travel to reach the target (i.e. the distance between fixation cross and pre-saccadically colored object). We then calculated saccade gain change GC with the following equation to obtain a measure of adaptation in percent:

(1) $$GC=\frac{G-{\bar{G}}_{pre}}{{\bar{G}}_{pre}}\times \;100$$

In this equation, ${\bar{G}}_{pre}$ is the average gain of the saccades in the pre-adaptation baseline trials. Secondary saccades were assessed during the adaptation procedure and included if they followed a primary saccade that landed between the pre- and post-saccadically colored object (conditions array shift and color shift) or between the initial and shifted position of the colored object (condition array shift with color swap). This was done in order to evaluate if the corrective saccades were made in the direction of the instructed saccade target or if gaze was corrected toward the wrong object within the array. Secondary saccades with an amplitude of more than 5 degrees in either direction, or with latencies of less than 100 ms and more than 600 ms, were discarded. After applying these criteria, valid corrective saccades were performed in 38.56% of the adaptation trials and included in the data analysis.

We computed mixed analyses of variance with the between-subjects factor instruction and the within-subjects factor feedback (2 × 3 ANOVA) on gain change, saccade latency and secondary saccade characteristics. Levene's test was used to assure homoscedasticity and Mauchly's test was performed to evaluate whether the assumption of sphericity was met. If the assumption of sphericity was violated, we applied the Greenhouse-Geisser correction. If the distribution of the residuals deviated from normality or homoscedasticity was not given, we additionally performed a robust ANOVA on the 20% trimmed median with the WRS2 package (Mair & Wilcox, 2020) to back up the results. If appropriate, Bonferroni-Holm corrected post hoc *t*-tests with an alpha level of 0.05 followed the ANOVA. If the data distribution was non-normal, Wilcoxon signed-rank tests were performed instead. If, for unpaired *t*-tests, homoscedasticity was not given, we used Welch's *t*-test. When we aimed at demonstrating that there was no difference in saccade gain change between the different conditions, we performed additional Bayesian *t*-tests to compare the evidence for the presence of an effect with the evidence for the absence of an effect. We chose a Cauchy prior centered around zero with a width parameter of 0.707, because we were interested in assessing the evidence for the absence of an effect. Additionally, we performed a robustness check and calculated the Bayes factors with a prior width of 1 and 1.41. Bayes factors were computed with the Bayes Factor package (Morey & Rouder, 2018).

Data analysis was conducted with MatLab (version R2018a; The Mathworks, Natick, MA, USA) and R (version 4.0.2; R Development Core Team, 2020).

## Results

### Gain change

**Figure 2.**
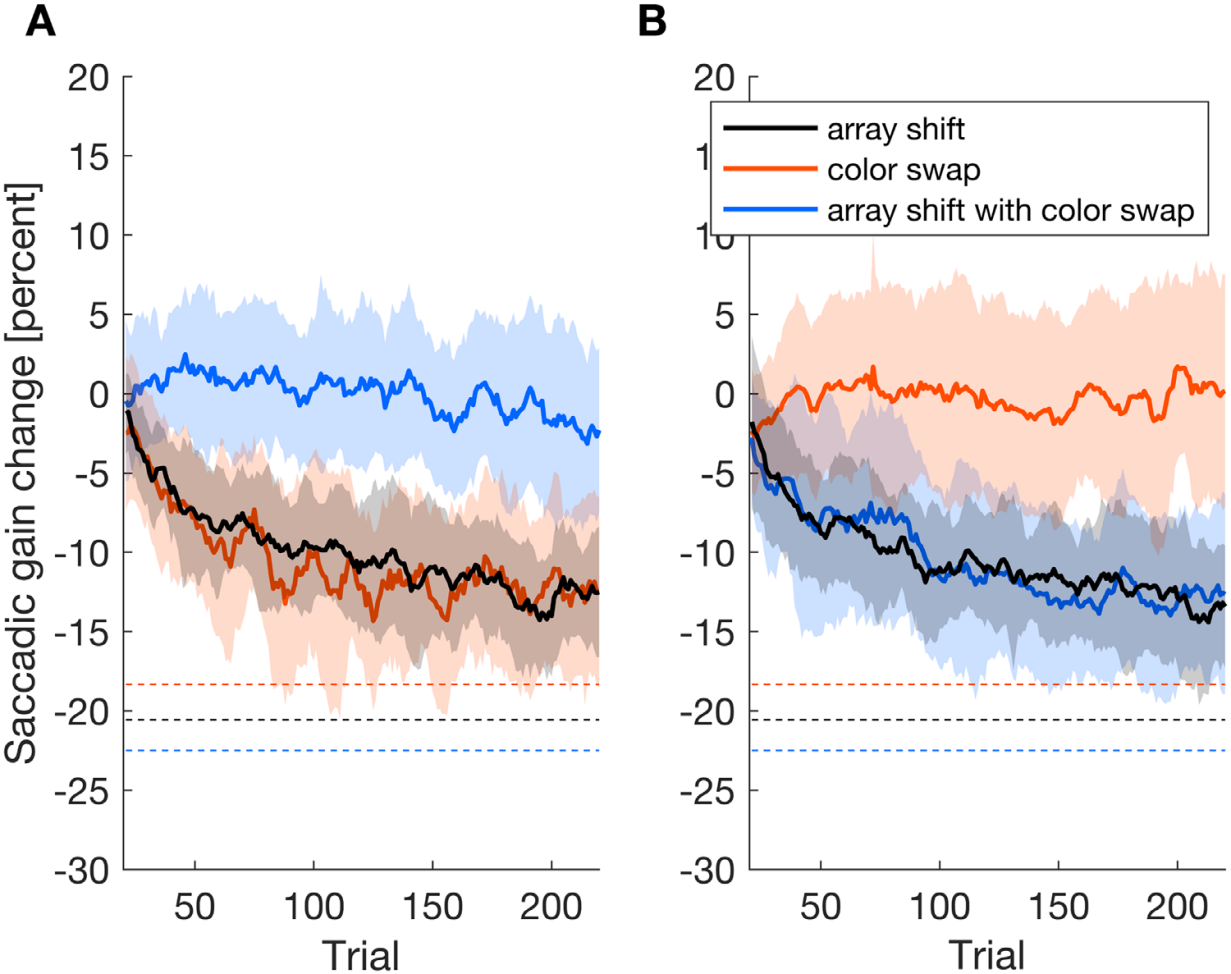
Moving average (window size = 10 trials) of the saccade gain change during the adaptation phase for the color instruction (**A**) and the position instruction (**B**) for the conditions array shift black) color swap (orange) and array shift with color swap (blue). The shaded areas indicate standard deviations. The dashed lines illustrate the change in saccadic gain that would mark complete adaptation to the intra-saccadic shift of the object array. Note that complete adjustment to the intra-saccadic manipulation corresponds to different levels of gain change in the different feedback conditions, since, during the adaptation procedure, the saccades were made toward a different subset of objects in each feedback condition while the intra-saccadic shift was always 2 degrees.

We aimed to investigate if participants are able to use information within the post-saccadic feedback flexibly to adjust their saccade motor performance to meet task demands. Thus, the development of saccade gain change throughout the experiment was assessed for both the instruction to look at the colored object (Figure 2A) and the instruction to look at the object at a particular position within the array (Figure 2B).

During the array shift condition (black lines), in which the sources of information contained in the post-saccadic feedback were congruent and required a shortening of the saccade amplitude, saccade gain change followed the exponential learning curve typically observed in saccade adaptation experiments (Deubel, Wolf, & Hauske, 1986; Ethier, Zee, & Shadmehr, 2008a; Panouillères, Weiss, Urquizar, Salemme, Munoz, & Pélisson, 2009; Straube, Fuchs, Usher, & Robinson, 1997) for either instruction.

During the color swap condition (orange lines), during which the information sources within the post-saccadic image were incongruent regarding the correct target object, the adaptation pattern was dependent on the instruction: for the instruction to look at the colored object, saccade gain decreased throughout the adaptation trials despite the absence of an array shift. Participants gradually shortened their amplitude and thus brought it increasingly closer to the target object. For the instruction to look at the object at the position within the array that was initially indicated by the red color, saccade gain remained constant throughout the adaptation procedure and the eye thus continued to land on the correct target object within the array despite the color swap. It is especially remarkable that saccade gain in the color swap condition decreased in parallel to the control condition of array shift although the object array in the color swap condition did not change position.

During the condition array shift with color swap (blue lines), during which the post-saccadic information was also incongruent, the pattern of gain change was again dependent on the instruction. For the instruction to look at the colored object, saccade metrics were maintained throughout the adaptation procedure despite the array shift against the saccade direction (i.e. the eye landed on the red object and the action goal was accomplished). For the instruction to look at the object at the initially specified position within the array, saccade amplitude was shortened during the adaptation procedure and the eye was brought increasingly closer to the correct goal.

**Figure 3.**
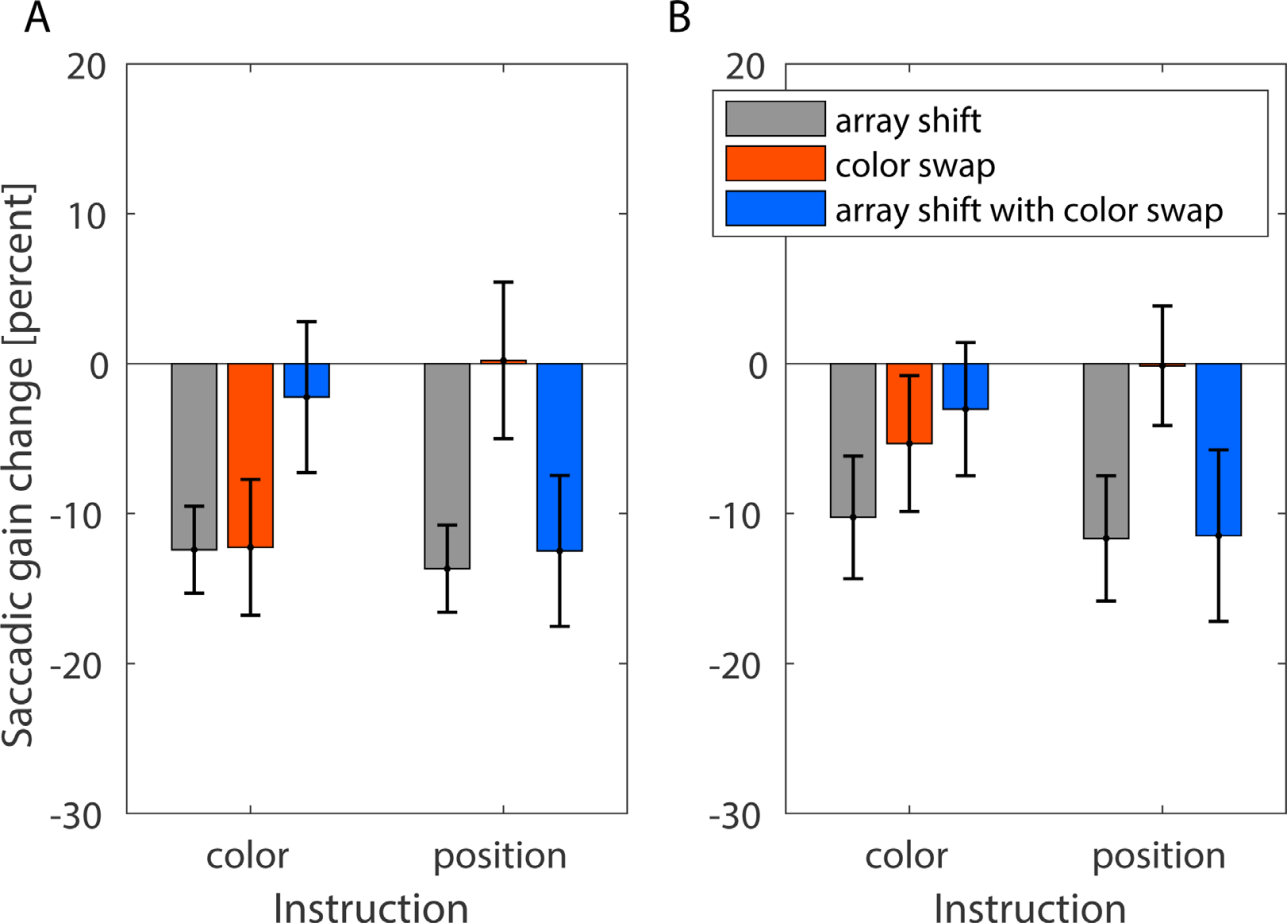
Average saccade gain change during late-adaptation (**A**) and during no-feedback post-adaptation trials (**B**) for both the instruction to look at the colored object (color) and the instruction to look at the predefined position within the object array (position) in the conditions array shift (black), color swap (orange) and array shift with color swap (blue). The error bars indicate standard deviations.

We assessed saccade gain change during late adaptation (trials 201:220) and during post-adaptation no-feedback trials (221:240). During late adaptation and following the instruction to look at the colored object, the average saccade gain change was –12.405% (*SD* = 2.90%), –12.25% (*SD* = 5.219%), and –2.231% (*SD* = 5.033%) for the conditions array shift, color swap, and array shift with color swap, respectively (Figure 3A).

For the instruction to look at the object at position x, the average saccade gain change was –13.672% (*SD* = 3.778%), 0.216% (*SD* = 6.61%), and –12.489% (*SD* = 4.702%) in the conditions array shift, color swap, and array shift with color swap. A mixed ANOVA with the within-subjects factor post-saccadic feedback (array shift, color swap, and array shift with color swap) and the between-subjects factor instruction (colored object and position) showed no significant main effect for the instruction type, *F*(1, 34) = 0.104, *p* = 0.749, η*^2^_p_ =* 0.003), but the main effect of post-saccadic feedback was significant (*F*(1.587, 53.944) = 17.629, *p* < 0.001, η*^2^_p_* = 0.395) and so was the interaction between feedback and instruction (*F*(1.587, 53.944) = 52.380, *p* < 0.001, η*^2^_p_* = 0.606). These results indicate that the instruction moderates the influence of post-saccadic feedback on the development of oculomotor performance.

For the array shift condition, during which the feedback was unambiguous and both instructions implied the same form of adjustment, no difference in gain change occurred (*t*(34) = 1.129, *p* = 0.267, *d* = 0.376, two-sided *t*-test, *BF_01_* = 1.894 [2.376, 3.106]).

In the color swap condition, gain change was significantly different for both instruction types (*t*(34) = –6.280, *p* < 0.001, *d* = 2.093, two-sided *t*-test) and the same applies to the array shift with color swap condition (*t*(34) = 6.319, *p* < 0.001, *d* = 2.106, two-sided *t*-test). Thus, post hoc tests confirmed that the same post-saccadic feedback can be used flexibly to accomplish different action goals.

During post-adaptation trials, the feedback was turned off upon saccade onset and participants did not receive feedback regarding their saccade accuracy. Thus, these trials serve to assess the after effects of adaptation that persist even though the feedback was removed. For the instruction to look at the colored object, the average saccade gain change was –10.25% (*SD* = 4.094%), –5.33% (*SD* = 4.529%) and –3.04% (*SD* = 4.40%) for the array shift, the color swap, and the array shift with color swap condition, respectively. For the instruction to look at the object at a particular position, the average gain change was –11.65% (*SD* = 4.176%) in the array shift condition, –0.141% (*SD* = 3.991%) in the color swap condition, and –11.47% (*SD* = 5.719%) in the array shift with color swap condition (Figure 3B). The 2 × 3 mixed ANOVA calculated on the post-adaptation data yielded the same pattern of results as the for the late-adaptation data. The main effect of instruction was not significant (*F*(1,34) = 2.484, *p* = 0.124, η*^2^_p_* = 0.068), but the main effect of feedback (*F*(2,68) = 34.481, *p* < 0.001, η*^2^_p_ =* 0.504) as well as the interaction between feedback and instruction (*F*(2,68) = 23.633, *p* < 0.001, η*^2^_p_* = 0.410) were significant. This pattern of results not only confirms that the use of the post-saccadic feedback depends on the action goal, but also shows that after effects, indicating implicit learning, show the same modulatory effect of instruction on the use of post-saccadic feedback.

Post hoc tests confirmed that task demands, here defined by type of instruction, in the presence of ambiguous post-saccadic information, determined which source of information is used to guide motor learning. Whereas for the array shift condition, in which post-saccadic information was congruent during the adaptation procedure, both instructions led to the same amount of implicit learning (*t*(34) = 1.017, *p* = 0.316, *d* = 0.339, two-sided *t*-test, *BF_01_* = 2.079 [2.632, 3.460]), for the color swap condition, more gain change was maintained following the instruction color than the instruction position (*t*(34) = –3.643, *p* = 0.005, *d* = 1.214, two-sided *t*-test) and for the array shift with color swap condition, less gain change was maintained following the instruction color than following the instruction position (*t*(34) = 4.944, *p* < 0.001, *d* = 1.648, two-sided *t*-test).

We also investigated if incongruent information within the post-saccadic visual feedback attenuated learning (i.e. if it interfered with the adjustment of saccade gain to the action goal). Therefore, we compared gain change obtained in the incongruent conditions (color swap, array shift with color swap) with gain change obtained in the congruent condition (array shift), that served as control condition. We did this separately for late-adaptation and post-adaptation trials. For the instruction to look at the colored object, the adjustment of saccade gain was equally strong in the array shift and in the color swap condition (*t*(17) = 0.111, *p* = 0.913, *d* = 0.026, two-sided *t*-test, *BF_01_* = 3.096 [4.098, 5.556]), which indicates that the incongruent position of the target object within the object array did not interfere with the required adjustment of saccade amplitude. The change to saccade gain in the array shift with color swap condition was less pronounced than in the array shift condition (*t*(17) = 8.096, *p* < 0.001, *d* = 1.908, two-sided *t*-test) and did not deviate significantly from zero (*t*(17) = –1.881, *p* = 0.077, *d* = 0.443, two-sided *t*-test, *BF_01_* = 0.971 [1.891, 1.536]), indicating no substantial development of gain change throughout the adaptation phase. Thus, participants adjusted their saccade motor performance to the goal without interference from incongruent post-saccadic information about the object position within the array. For the position instruction, the adjustment of gain was equally strong in the array shift with color swap condition as in the array shift condition (*t*(17) = 0.875, *p* = 0.394, *d* = 0.206, two-sided *t*-test, *BF_01_* = 2.375 [3.049, 4.049]), and thus unaffected by incongruent post-saccadic color information. In the color swap condition, saccade gain change was less pronounced than in the array shift condition (t(17) = 9.567, p < 0.001, *d* = 2.255, two-sided *t*-test) and remained around zero throughout the adaptation procedure (*t*(34) = 0.139, *p* = 0.891, *d* = 0.033, two-sided *t*-test, *BF_01_* = 4.082 [5.525, 7.634]), indicating that participants maintained their original saccade gain and continued to meet their goal without experiencing any interference from incongruent color information.

We then assessed whether incongruent post-saccadic information might have affected the after effect (i.e. whether the saccade gain change that carried over to the no-feedback trials reflects implicit learning rather than just a strategic adjustment to an error signal). For this, we conducted the same analysis for the post-adaptation trials. For the instruction to look at the colored object, saccade gain change in the array shift with color swap condition remained smaller than gain change in the congruent array shift condition (*t*(17) = 7.489, *p* < 0.001, *d* = 1.765, two-sided *t*-test), but some significant after-effect emerged (*t*(17) = -2.900, *p* = 0.040, *d* = 0.684, two-sided *t*-test, *BF_01_* = 0.1887 [0.210, 0.253]). This indicates that incongruent information about the relative position, induced by the shift of the object array, led to an implicit shortening of saccade amplitude (i.e. learning). The effect of this implicit learning process might have been masked by the execution of a strategically planned saccade that would steer the eye toward the post-saccadically colored object and that became only visible once the feedback was removed and the strategy no longer applied. Thus, the incongruent spatial information appears to have affected saccade execution latently.

In the color swap condition, less gain change was carried over to the no-feedback trials than in the array shift condition (*t*(17) = 4.058, *p* = 0.005, *d* = 0.957, two-sided *t*-test). This decrease in gain change could either be due to an interference effect of incongruent position information, or, more likely, be a characteristic of adaptation without spatial prediction error. For this form of saccade adaptation, an explicit strategic component might play a more important role and it is known to produce smaller after effects than conventional adaptation (Schütz et al., 2014).

For the position instruction, the pattern of results from the late-adaptation trials was confirmed in the no-feedback trials. In the array shift with color swap condition, as much gain change was transferred to the no-feedback trials as in the array shift condition (*t*(17) = 0.112, *p* = 0.912, *d* = 0.026, two-sided *t*-test, *BF_01_* = 3.096 [4.098, 5.556]), indicating that no interference occurred. Thus, the incongruent color information did not attenuate adaptive adjustment of the saccadic amplitude. In the color swap condition, saccade gain change was substantially less pronounced than during the array shift condition (*t*(17) = 8.344*, p* < 0.001, *d* = 1.967, two-sided *t*-test) and remained around zero (*t*(17) = –0.150, *p* = 0.883, *d* = 0.035, two-sided *t*-test, *BF_01_* = 4.082 [5.525, 7.634]). Thus, the incongruent color information did not interfere with maintaining a stable saccade amplitude throughout the adaptation procedure.

### Primary saccade latency

We also assessed if the different feedback types or instructions affected saccade latency and calculated a mixed ANOVA on both the late-adaptation and the post-adaptation data. During late-adaptation trials, average latencies for the instruction to look at the colored object were 238.001 ms (*SD* = 58.608 ms), 229.493 ms (*SD* = 52.502 ms), and 228.354 ms (*SD* = 33.973 ms) for the array shift, the color swap, and the array shift with color swap condition, respectively. For the position instruction, the average latency was 227.314 ms (*SD* = 34.234 ms) in the array shift condition, 247.486 ms (*SD* = 66.068 ms) in the color swap condition and 233.886 ms (*SD* = 35.396 ms) in the array shift with color swap condition. Neither the main effect of instruction (*F*(1,34) = 0.114, *p* = 0.738, η*^2^_p_* = 0.003), nor the main effect of feedback type (*F*(1.683, 57.233) = 0.398, *p* = 0.638, η*^2^_p_* = 0.012), nor their interaction (*F*(1.683, 57.233) = 1.361, *p* = 0.263, η*^2^_p_* = 0.038) were significant. Because the residuals showed some moderate deviation from normality, we calculated an additional robust mixed ANOVA on the 20% trimmed median (Mair & Wilcox, 2020), which confirmed the results of the original ANOVA (all *F* < 0.974, all *p* > 0.399).

During the no-feedback post-adaptation trials, average latencies for the instruction to look at the colored object were 220.357 ms (*SD* = 27.188 ms), 221.281 ms (*SD* = 32.525 ms), and 218.328 ms (*SD* = 28.561 ms) for the conditions array shift, color swap, and array shift with color swap, respectively. For the position instruction, primary saccade latency was 214.163 ms (*SD* = 27.418 ms) in the array shift condition, 217.842 ms (*SD* = 31.324 ms) in the color swap condition, and 214.163 ms (*SD* = 27.418 ms) 226.464 ms (*SD* = 35.848 ms) in the array shift with color swap condition. As during the late-adaptation trials, neither the instruction (*F*(1,34) =0.004, *p* = 0.947, η*^2^_p_* < 0.001), nor the feedback (*F*(2,68) = 0.368, *p* = 0.694, η*^2^_p_* = 0.011), nor their interaction affected the saccade latency (*F*(2,68) = 0.803, *p* = 0.452, η*^2^_p_* = 0.023). The absence of an effect of feedback type or instruction is not surprising because the participants in our study performed overlap saccades and thus had ample time to prepare their eye movement toward the target object before they received the go-signal for saccade execution.

### Secondary saccades

**Figure 4.**
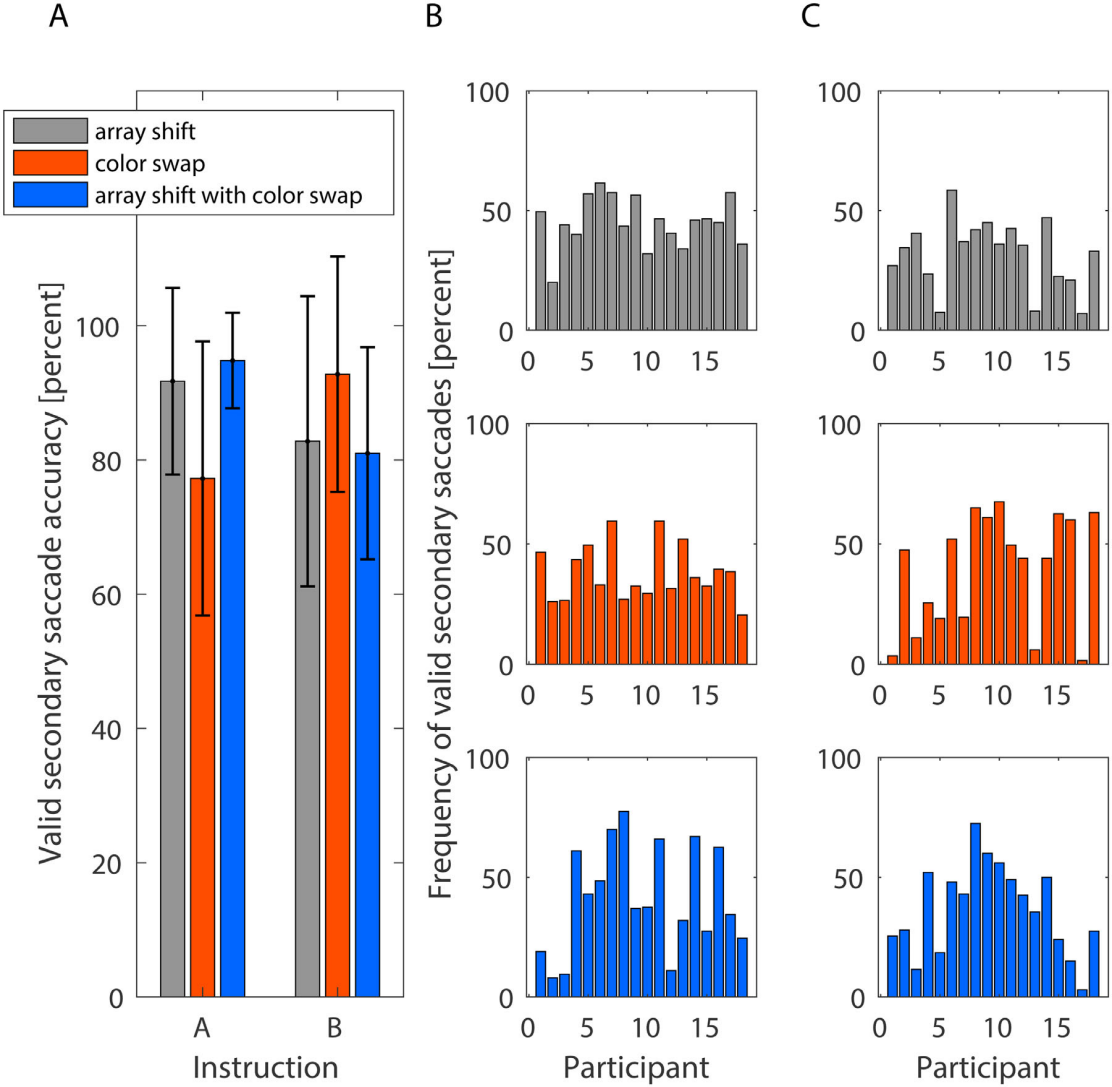
Secondary saccades that follow a primary saccade that has landed between the conflicting target positions during the adaptation phase. (**A**) The accuracy of the secondary saccades, that is, the percentage of all valid secondary saccades that were aimed toward the target object in the array shift (grey), the color swap (red), and the array shift with color swap (blue) condition, depicted separately for the color instruction and the position instruction. The error bars indicate standard deviations. (**B**) The frequency of valid secondary saccades for each participant during the adaptation procedure for the color instruction. (**C**) The frequency of valid corrective saccades for each participant during the adaptation procedure for the position instruction.

After assessing the influence of instruction and feedback type on primary saccade gain and latency, we investigated secondary saccade characteristics, such as latency and accuracy. Secondary saccades are not necessary for adaptation to occur (Wallman & Fuchs, 1998), but are typically made to shift the fovea from the primary saccade end point to the saccade target object. Thus, we assessed whether participants corrected their gaze to the object that was, by instruction, defined as target object. For these analyses, we only included secondary saccades that followed a primary saccade that landed either between the two conflicting post-saccadic targets (conditions array shift with color swap and color swap) or between the pre- and post-saccadic target position of the saccade target (array shift). In this way, we could analyze whether the gaze correction that followed the primary saccade occurred in the right direction (i.e. toward the target object). Saccades that were aimed toward the defined target object were considered accurate. The average accuracy of secondary saccades in the different conditions is depicted in Figure 4A. For the instruction to look at the colored object, the average accuracy was 91.74% (*SD* = 13.90%) in the array shift condition, 77.24% (*SD* = 20.42%) in the color swap condition and 94.81% (*SD* = 7.11%) in the array shift with color swap condition. For the position instruction, the average accuracy was 82.79% (*SD* = 21.61%), 92.78% (*SD* = 17.54%), and 80.10% (*SD* = 15.81%) in the conditions array shift, color swap, and array shift with color swap, respectively. However, note that there are participants who made few primary saccades that landed between the two conflicting post-saccadic targets and that were additionally followed by secondary saccades that matched our inclusion criteria. Thus, for some participants only few secondary saccades entered our further analysis (Figures 4B, 4C). We calculated a mixed ANOVA on the accuracy of the secondary saccades and found neither a significant main effect of instruction (*F*(1, 34) = 0.529, *p* = 0.472, η*^2^_p_* = 0.015) nor feedback (*F*(2, 68) = 0.304, *p* = 0.739, η*^2^_p_* = 0.009). The interaction was significant (*F*(2, 68) = 8.144, *p* < 0.001, η*^2^_p_* = 0.193). Because the residuals showed some moderate deviation from normality and the data were heteroscedastic, we calculated an additional robust mixed ANOVA on the 20% trimmed median (Mair & Wilcox, 2020), which confirmed the pattern of results (instruction: *F*(1, 21.998) = 0.226, *p* = 0.639; feedback: *F*(2, 16.689) = 0.633, *p* = 0.543; interaction: *F*(2, 16.689) = 7.989, *p* = 0.004).

Post hoc tests showed no difference in accuracy between both instructions in the array shift (*t*(34) = 1.477, *p* = 0.149, *d* = 0.492, two-sided *t*-test) or the color swap condition (*t*(34) = -2.448, *p* = 0.118, *d* = 0.816, two-sided *t*-test), but during the array shift with color swap condition, secondary saccade accuracy was higher for the instruction to look at the colored object than for the position instruction (*t*(23.614) = 3.381, *p* = 0.020, *d* = 1.127, two-sided Welch's *t*-test). When assessing possible interference of incongruent information on secondary saccade accuracy, we found no evidence of lower accuracy in the incongruent conditions (color swap: *p* = 0.064, *r* = 0.63, one-sided Wilcoxon signed-rank test; array shift with color swap: *p* = 0.882, *r* = 0.320, Wilcoxon signed-rank test) than in the congruent array shift condition for the instruction to look at the colored object. However, the difference in accuracy between the array shift with color swap and the color swap condition was significant (*t*(17) = 3.617, *p* = 0.019, *d* = 0.853, two-sided *t*-test). When instructed to look at the object at the specified position, secondary saccade accuracy was not lower in the incongruent conditions (color swap: *p* = 0.977, *r* = 0.542, one-sided Wilcoxon signed-rank test; array shift with color swap: *p* = 0.224, *r* = 0.216, one-sided Wilcoxon signed-rank test) than in the congruent condition array shift. There was no significant difference in secondary saccade accuracy between the array shift with color swap and color swap condition (*p* = 0.118, *d* = 0.634, two-sided Wilcoxon signed-rank test). Overall, the results do not suggest that incongruent post-saccadic information weakened the ability to perform accurate corrective saccades. It appears that participants recognized the saccade target, defined by either the feature dimension color or by position, after the primary saccade and then initiated the gaze correction successfully. The results suggest that gaze correction was particularly stable in the array shift with color swap condition when participants followed the instruction to look at the colored object, with accuracy being higher than for the same post-saccadic feedback when following the position instruction, and accuracy being also higher than in the color swap condition following the instruction to look at the colored object. However, this result should be treated with caution because, firstly, the origin of this advantage is unclear and, secondly, some participants contributed only little data to this analysis.

**Figure 5.**
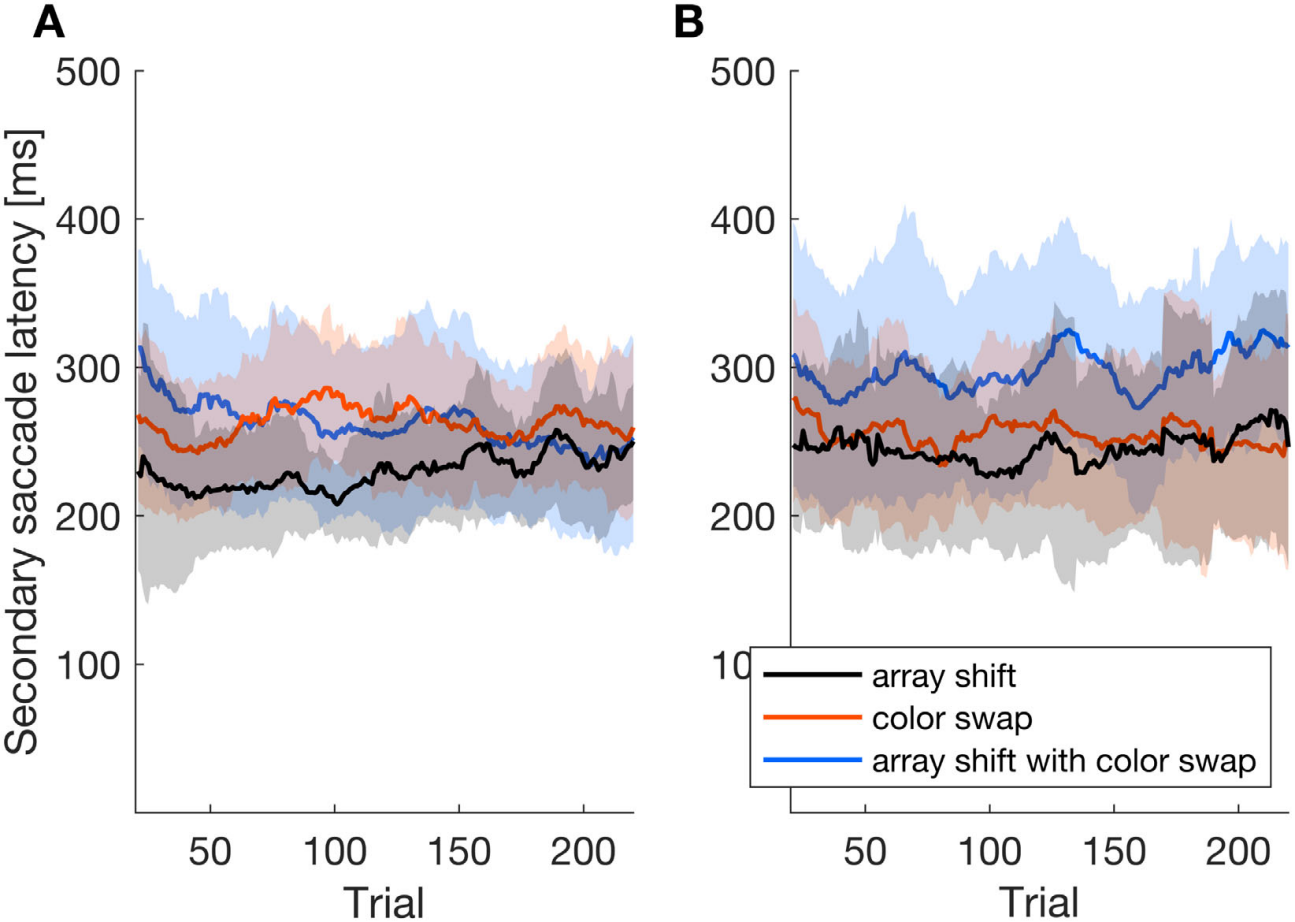
Moving average (window size = 20 trials) of the valid secondary saccade latency throughout the adaptation procedure for the color instruction (**A**) and the position instruction (**B**) for the conditions array shift black), color swap (orange) and array shift with color swap (blue). The shaded areas indicate standard deviations.

We also investigated the influence of post-saccadic feedback and instruction type on the average secondary saccade latency throughout the adaptation procedure (Figure 5). The mixed ANOVA showed a significant main effect of feedback (*F*(2,68) = 10.818, *p* < 0.001, η*^2^_p_* = 0.241) whereas neither the main effect of instruction (*F*(1,34) = 1.732, *p* = 0.197, η*^2^_p_* = 0.048) nor the interaction between feedback and instruction (*F*(2,68) = 1.686, *p* = 0.193, η*^2^_p_* = 0.047) had a significant impact on the secondary saccade latency. In order to further investigate the effect of post-saccadic information and also a possible interference of conflicting target objects for gaze correction, we calculated post-hoc t-tests. For the color instruction, the secondary saccade latency was larger in the color swap than in the array shift condition (array shift: *M* = 263.79 ms, *SD* = 33.377 ms; color swap: *M* = 228.65 ms, *SD* = 29.13 ms; *t*(17) = 3.686, *p* = 0.005, *d* = 0.869, one-sided *t*-test). The secondary saccade latency in the array shift with color swap condition (*M* = 263.71 ms, *SD* = 49.95 ms) was also higher than in the array shift condition (*t*(17) = 2.580, *p* = 0.039, *d* = 0.608, one-sided *t*-test), whereas the difference between the two incongruent conditions array shift with color swap and color swap was not significant (*t*(17) = –0.006, *p* = 0.995, *d* = 0.001, two-sided *t*-test). Thus, for the instruction to look at the colored object, secondary saccade latency was higher when there were conflicting targets for gaze correction.

For the position instruction, secondary saccade latency was higher in the array shift with color swap condition than in the array shift condition (array shift with color swap: *M* = 293.28 ms, *SD* = 59.92 ms; array shift: *M* = 243.07 ms, *SD* = 40.59 ms; *t*(17) = 3.440, *p* = 0.008, *d* = 0.811,one-sided *t*-test). The color swap condition (*M* = 259.51 ms, *SD* = 43.64 ms) did not evoke longer latencies than the array shift condition (*t*(17) = 1.350, *p* = 0.097, *d* = 0.318, one-sided *t*-test) and no difference in secondary saccade latency occurred between the incongruent color swap with array shift and the color swap condition (*t*(17) = 2.209, *p* = 0.124, *d* = 0.521, two-sided *t*-test). Thus, for the position instruction, the interference effect of incongruent post-saccadic information did not show in the color swap condition.

## Discussion

Humans constantly recalibrate their oculomotor behavior since precise eye movements are vital for clear vision and for interacting with objects in the environment. Saccade adaptation, the process that ensures the accuracy of saccadic eye movements, was long considered a low-level learning mechanism based on bottom-up visual feedback from the post-saccadic image. Yet, precisely guiding our eye movements and adjusting the saccade metrics is often a necessary means to achieve behavioral goals, which suggests a contribution of top-down processes to saccade adaptation. In the present study, we demonstrated that humans can flexibly select information sources within the post-saccadic image to adjust their oculomotor behavior to task demands. Our participants were instructed to either look at the colored object within an object array or to look at an object at a predefined position within the array (e.g. the object at the second position). Participants then completed three conditions, each with a different manipulation of the post-saccadic feedback. We investigated whether our participants were able to either modify or maintain their saccade metrics to meet different task demands with the same visual feedback, which could present conflicting post-saccadic information regarding the correct target object. We compared the oculomotor behavior in these incongruent conditions with a control condition, in which the post-saccadic image provided congruent information.

We report three main findings: first, our participants were able to use the different sources of information in the post-saccadic visual feedback flexibly to adapt their saccade gain to current goals. Although they were presented with the same post-saccadic image, our participants’ primary saccade gain differed between groups and thus was dependent on the instructions (i.e. the task). Second, saccadic adaptation could be induced without the spatial error that occurs after intra-saccadic displacement of the target, or, as presented here, without a shift of the object array. Third, control of saccade gain was not subject to any interference of incongruent color information. For incongruent spatial information this was less clear.

Possible interference of incongruent color and position information was assessed for conditions requiring to maintain a stable gain and for conditions requiring adaptation of saccade gain. When the participants were instructed to look at an object at a certain position within the array and this task required a shortening of the saccade amplitude, participants adjusted their saccade behavior in the same manner regardless of whether the post-saccadic image provided congruent or incongruent color information. Participants were also able to maintain a stable saccade gain throughout the adaptation procedure and the subsequent no-feedback trials, when this was required to continuously bring their eye to the target object. Consequently, incongruent color information did not prevent successful performance of the task. Whether there was interference from incongruent position information cannot be answered easily. Following the instruction to look at the colored object, participants adjusted their saccade behavior in parallel to the control condition with congruent post-saccadic information. However, during the post-adaptation no-feedback trials, saccade gain showed a significantly weaker after effect, indicating less learning. This result is consistent with a previous study by Schütz et al. (2014), who induced a gradual adjustment of saccade behavior without a spatial error and also reported a substantial, but reduced after effect compared to adaptation following an intra-saccadic target displacement. Therefore, it is likely that the decrease in adaptation between late-adaption and no-feedback trials is not due to incongruent post-saccadic information, but rather a typical feature of adaptation without spatial error. However, we cannot rule out the possibility that the incongruent post-saccadic position information might have counteracted the learning process during the adaptation process and that, for this reason, once the intra-saccadic manipulation was removed, weaker adaptation was observed. When participants maintained a constant saccade gain, adaptation to the position error had to be inhibited in order to look at the post-saccadically colored object. In this condition, saccade amplitude remained constant at the end of the adaptation period but changes to the saccade gain emerged during the following post-adaptation trials. Thus, the inhibition of significant changes to saccade gain during the adaptation procedure was successful, but when the post-saccadic feedback was removed during the saccade, significant gain change in direction of the array shift occurred. This suggests that the intra-saccadic manipulation and the associated spatial error led to latent learning throughout the adaptation procedure, that was masked by strategic oculomotor behavior that was performed until the array was no longer shifted and participants deemed it no longer necessary. In addition, the interplay of a slow and a fast learning process, with strong and weak retention, respectively, could also have contributed to this result. It has been shown that saccade adaptation relies on both processes (Ethier, Zee, & Shadmehr, 2008b) and it might be possible that, in the face of incongruent post-saccadic information, the intra-saccadic shift of the object array has led to slow and persistent learning, the consequences of which remained hidden due to fast adjustment of the saccade to the color information. Once the manipulation was removed, the fast component might have quickly been forgotten, resulting in a stronger change in saccade gain in the post- than the late-adaptation trials in the direction of the array shift. Yet, this remains speculative, especially because the learning curves throughout the adaptation procedure did not indicate any interference from a parallel learning process. Consistent with previous research, a discrepancy between expected and actual sensory consequences of a movement can induce adaptation, even when it interferes with task demands (Mazzoni & Krakauer, 2006). Consequently, the spatial error might not be the only information that can be used for oculomotor learning. However, when a spatial error occurs, it appears to be difficult to ignore, probably because the planning of saccadic eye movements depends heavily on spatial information.

Secondary saccades, the eye movements that follow the primary saccade and bring the eye closer to the target in case it was missed, were remarkably accurate, regardless of congruent or incongruent post-saccadic information. The high accuracy indicates that knowledge about the defining feature of the target facilitates object recognition and gaze correction. Neither incongruent color nor position information weakened our participants’ ability to perform corrective saccades to the right object within the array. Nevertheless, when the available post-saccadic information regarding the saccade target was manipulated and not all features of the post-saccadic target object corresponded to those stored in visual working memory, the secondary saccade latency increased. This is in line with the finding of Richard, Luck, & Hollingworth (2008) that incongruent position or color information leads to slower and less accurate gaze corrections. This effect of incongruent information might be due the increased cognitive load associated with the higher difficulty of selecting the correct object.

Our results add to the growing evidence that task demands, pending actions, and intentions can modulate saccade adaptation in a top-down manner (Heins et al., 2019; Madelain et al., 2011; Schütz et al., 2014; Schütz & Souto, 2015). In this view, target selection is the process that drives post-saccadic error evaluation and saccade adaptation (Schütz & Souto, 2015; Wagner et al., 2021). In our study, the instruction provided information about the defining feature of the target object and this feature information appears to have controlled our participants’ target selection and thereby oculomotor behavior. It is noteworthy that very different feature dimensions, here color or position, can be voluntarily selected and used to guide oculomotor learning.

Tasks and intentions are known to render certain objects or object properties relevant over others (Bekkering & Neggers, 2002; Fagioli, Hommel, & Schubotz, 2007; for a review see Heuer, Ohl, & Rolfs, 2020). Planning an action, here an eye movement, might have facilitated the perception of the task-relevant over the task-irrelevant feature dimension. Knowing the target-defining feature dimension in advance can increase the assigned weight for that dimension, enhance its perception and thereby speed detection of and reaction to a target (Müller, Reimann, & Krummenacher, 2003; for a review see Memelink & Hommel, 2013). It appears that, in our study, the instruction and subsequent intention to reach the target object with an eye movement primed task-relevant feature dimensions and thereby facilitated the selection of the relevant information within the post-saccadic image. In this way, our participants not only learned an eye-movement behavior that brought their primary saccade landing point increasingly close to the post-saccadic position of the target object, but they also corrected gaze efficiently in case of an error.

The changes in saccade amplitude following different error signals developed gradually over the course of the experiment, consistent with typical error-based learning (Straube et al., 1997). In addition, significant after effects occurred both for the conventional adaptation following a shift of the object array and, to a smaller degree, for the task of reaching the color-defined target object within the stationary array. The slow time course and the persistence of the changes in saccade gain indicate that the observed changes in oculomotor behavior are not exclusively due to the use of a strategy but rather to a continuous learning process. By showing that task-related intentions determine which source of information within the post-saccadic visual input is selected to guide motor learning, this study further highlights the close interconnectedness of eye movements and action intentions in the natural world. It provides evidence for the high flexibility of the visual system by decoupling saccade adaptation from spatial error and attributing it to target selection. The visual image of the world we live in and interact with is rich, and so is the variety of signals we can use to adjust the motor command for our eyes not only to ever-changing conditions, but also to a wide variety of tasks.

## Conclusion

Our results suggest that target selection is the mechanisms behind the error evaluation following a saccade. In this view, saccade adaptation not only reduces bottom-up spatial errors, but also adjusts our oculomotor behavior to current task demands, flexibly using the relevant sources of information within the visual image.
